# Anti-MSLN chimeric antigen receptor-like NK cell therapy with tumor-penetrating capacity (uCAR-like NK) for solid tumors

**DOI:** 10.1038/s41392-025-02524-0

**Published:** 2025-12-23

**Authors:** Mengchao An, Ying Wang, Jie Shao, Siwen Wu, Jiayao Yan, Yuxiang Li, Liqing Zhong, Jingyi Guo, Tianran Chen, Manman Tian, Qin Liu, Rutian Li, Baorui Liu

**Affiliations:** 1 Department of Oncology, Nanjing Drum Tower Hospital Clinical College of Nanjing Medical University, Nanjing, China; 2 The Comprehensive Cancer Centre, Nanjing Drum Tower Hospital & Group’s Suqian Hospital, Affiliated Hospital of Medical School, Nanjing University, Nanjing, China

**Keywords:** Drug development, Immunotherapy

## Abstract

Although natural killer (NK) cells are endowed with intrinsic cytotoxicity, their therapeutic application often faces limitations because of their lack of tumor-specific targeting ability and limited ability to infiltrate solid tumors. To overcome these limitations, we developed anti-mesothelin (MSLN) uCAR-like NK cells, which are designed to enhance both the targeting specificity and tumor infiltration capacity, thereby improving the antitumor efficacy of NK cell-based therapies. We constructed, purified, and validated a tetravalent bispecific cell engager (MSLN×CD16A) via the SpyTag/SpyCatcher system. Cytokine-induced memory-like NK cells, induced by IL-12, IL-15, and IL-18, were precomplexed with MSLN×CD16A to generate anti-MSLN CAR-like NK cells. To further enhance tumor penetration, the tumor-penetrating peptide uCendR was integrated into the system to construct anti-MSLN uCAR-like NK cells. In vitro, anti-MSLN CAR-like NK cells demonstrated selective cytotoxicity against MSLN-positive tumor cells through stable binding with MSLN×CD16A while sparing MSLN-negative cells. In xenograft models bearing MSLN-positive tumors, anti-MSLN CAR-like NK cells exhibited significant antitumor activity, with favorable tolerability and no significant body weight loss or toxicity. Notably, anti-MSLN uCAR-like NK cells, which integrate a tumor-penetrating peptide, displayed enhanced intratumor penetration and superior therapeutic efficacy. Overall, this study establishes a modular, nongenetically engineered uCAR-like NK platform that couples targeted recognition with enhanced tissue access. These findings highlight the potential of anti-MSLN CAR-like NK cells, particularly uCAR-like NK cells with enhanced tumor penetration, as promising therapeutic strategies for MSLN-positive solid tumors and lay the foundation for future clinical applications.

## Introduction

Adoptive natural killer cell therapy has emerged as a safe and effective novel immunotherapeutic strategy, offering renewed hope for patients with cancer. NK cells, as critical components of the innate immune system, possess natural cytotoxic activity and can independently recognize and eliminate tumor cells, resulting in “natural killers”.^1^ Unlike T lymphocytes, NK cells do not rely on specific antigen recognition and are not restricted by MHC, enabling them to target and kill tumor cells with downregulated or absent MHC expression.^2^ Furthermore, NK cells exhibit good accessibility, a broad tumor antigen spectrum, and superior safety, with a lower incidence of adverse effects such as graft-versus-host disease (GvHD), cytokine release syndrome (CRS), and neurotoxicity.^3^ After brief activation with IL-12, IL-15, and IL-18, NK cells can differentiate into functionally enhanced cytokine-induced memory-like (CIML) NK cells, which exhibit increased proliferation, cytotoxicity, and prolonged in vivo persistence.^4–6^ Therefore, CIML NK cells serve as ideal carriers for CAR-NK cells, with the potential to further enhance antitumor activity. Despite the promising prospects of CAR-NK cells in treating hematological malignancies and solid tumors, the lack of effective gene transfection methods for primary NK cells remains a bottleneck in the development of this therapy.

NK cell engagers (NKCEs) provide a novel strategy for optimizing NK cell efficacy. NKCEs are typically bispecific or trispecific proteins composed of antigen-targeting domains (usually scFvs) derived from antibodies, with one domain targeting NK cell activation receptors (such as CD16A, NKG2D, etc.), and the other binding to specific tumor-associated antigens.^7,8^ By precomplexing NKCEs with adoptive NK cells, CAR-like NK cells can be constructed without genetic modification, enhancing the antitumor potential of the immune system and effectively initiating synergistic antitumor immune responses.^9–11^ The close interaction between NKCE and NK cells avoids the risks associated with gene-engineered CAR-NK cells, such as dose-limiting toxicity and malignant reactivation.^12^

Mesothelin (MSLN) is a cell surface glycoprotein that is highly overexpressed in various malignant tumors but is restricted to mesothelial cells in normal tissues, reducing the risk of off-target toxicity.^13,14^ Moreover, the expression of MSLN is associated with chemotherapy resistance, poor prognosis, and increased tumor aggressiveness and may facilitate tumor invasion and metastasis.^15^ These features make MSLN an attractive target for CAR-T and CAR-NK cell immunotherapy in solid tumors.

Tumor-penetrating peptides can effectively enhance drug infiltration in solid tumors. Currently, most penetrating peptides exert their effects through the C-end rule (CendR), where the C-terminal exposed R/K-X-X-R/K sequence (X represents any amino acid) specifically binds to neuropilin 1 (NRP-1), thereby increasing the permeability of the tumor vasculature and tissues and facilitating drug delivery.^16^ A tumor-penetrating peptide, uCendR (RPARSGR↓SAGGSVA, where ↓ denotes the cleavage site), which is based on the common cleavage motif of urokinase-type plasminogen activator (uPA), is cleaved by uPA, triggering its binding to cells expressing NRP-1.^17^ uPA, a tumor-associated serine protease, is overexpressed in most tumors but is expressed at low levels in normal tissues.^18^

**Fig. 1:**
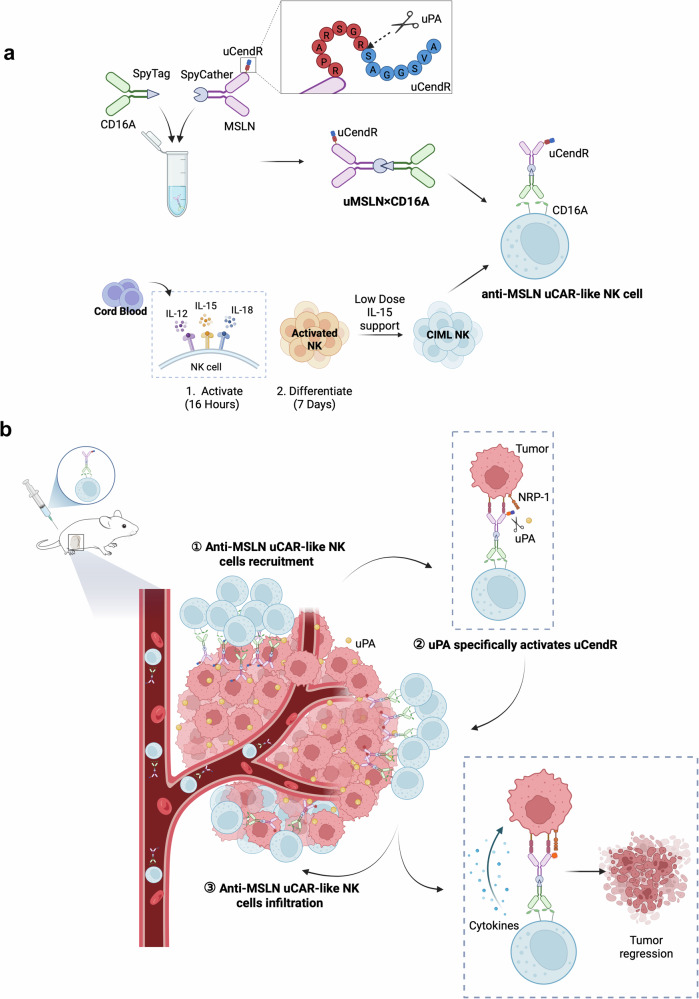
Schematic illustration of the antitumor effects of anti-MSLN uCAR-like NK cells in solid tumors. **a** (i) uCAR-like NK cell therapy is generated by arming CIML NK cells with a SpyTag/SpyCatcher-assembled bispecific engager (MSLN×CD16A) that incorporates a tumor-penetrating peptide (uCendR). **b** (ii) Upon systemic infusion, anti-MSLN uCAR-like NK cells preferentially accumulate in MSLN-positive tumors and initiate NK cell activation. (iii) Tumor-associated uPA specifically cleaves and activates uCendR, enabling it to bind NRP-1 on tumors and infiltrate MSLN-positive solid tumors, thereby promoting antitumor effects. (iv) In xenograft models, anti-MSLN uCAR-like NK cells accumulate within MSLN-positive tumors and lead to potent tumor regression with good tolerability. Figures were created with BioRender.com and SciDraw

In this study, we innovatively employed the SpyTag/SpyCatcher protein conjugation system derived from the *Streptococcus pyogenes* CnaB2 protein,^19,20^ which links SpyTag to CD16A DARPin and SpyCatcher to MSLN DARPin. The lysine (K31) in SpyCatcher reacts with aspartic acid (D117) in SpyTag to form a stable covalent bond, constructing the bispecific cell engager MSLN×CD16A. MSLN×CD16A was precomplexed with CIML NK cells in a membrane-modified manner to develop a novel CAR-like NK cell therapy. SpyTag/SpyCatcher acts as a “molecular superglue” and imparts modular universality to the CAR-like NK cell platform. To further enhance the immune infiltration of CAR-like NK cells in solid tumors, we linked the tumor-penetrating peptide uCendR to the MSLN end. This modification endows anti-MSLN uCAR-like NK cells with both targeting and tumor tissue penetration capabilities. We anticipate that anti-MSLN uCAR-like NK cells specifically accumulate in MSLN-positive tumor tissues, where the uCendR peptide is processed into active CendR peptides under the influence of uPA in the tumor microenvironment, triggering immune cells to penetrate deep into the tumor and enhancing the tumor-killing effects (Fig. 1). Our findings demonstrate that anti-MSLN uCAR-like NK cells not only possess exceptional tumor penetration and homing capabilities but also exhibit potent antitumor activity against MSLN-positive tumors, laying the foundation for their clinical application.

## Results

### Construction and characterization of the bispecific cell engager MSLN×CD16A

**Fig. 2:**
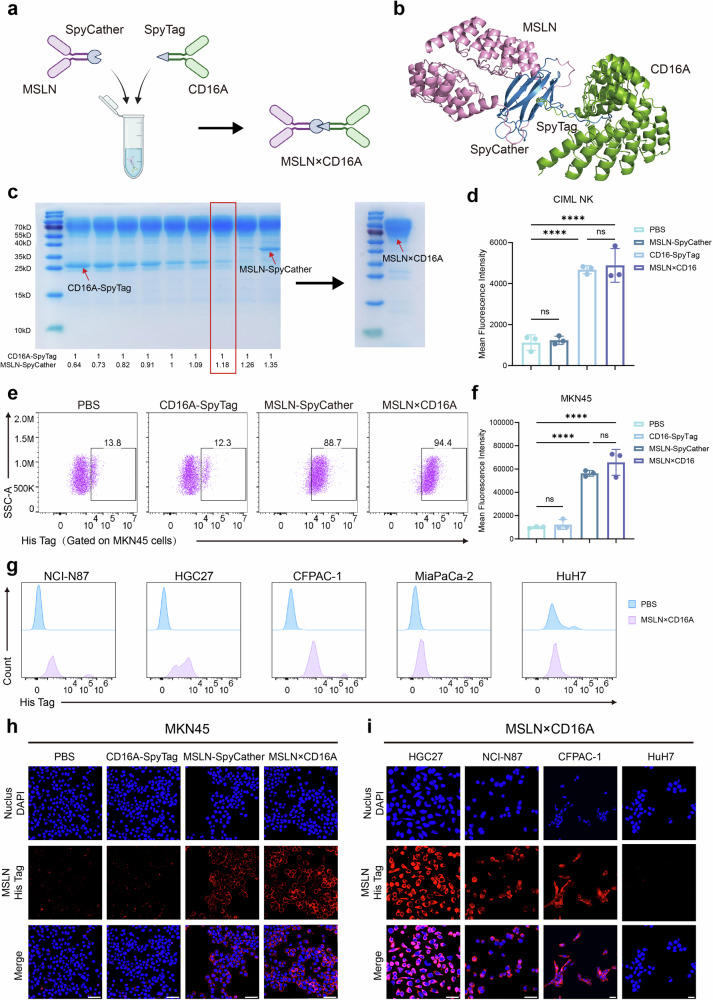
Synthesis and binding characteristics of MSLN×CD16A. **a** Schematic diagram of MSLN×CD16A synthesis. Through the SpyTag/SpyCatcher protein conjugation system, MSLN×CD16A was synthesized with a molecular ratio of 1:1 between the target end MSLN-SpyCatcher and the effector end CD16A-SpyTag. **b** Visualization of the MSLN×CD16A structure via AlphaFold and PyMOL. **c** The target end MSLN-SpyCatcher and the effect end CD16A-SpyTag were mixed and coupled at different ratios with a 1:1 molecular ratio as the baseline to determine the optimal reaction conditions (left figure), with the red box indicating the optimal ratio. The right figure shows the SDS‒PAGE results for MSLN×CD16A. **d** Flow cytometry results demonstrating the binding of MSLN×CD16A to CIML NK cells. **e** Representative flow cytometry images of CD16A-SpyTag, MSLN-SpyCatcher, and MSLN×CD16A binding to MSLN-positive tumor cells (MKN45). **f** Mean fluorescence intensity of MKN45 in (**e**). **g** Binding of MSLN×CD16A to MSLN-positive (NCI-N87, HGC27, and CFPAC-1) and MSLN-negative (MiaPaCa-2, HuH-7) tumor cells. **h** Immunofluorescence staining to detect the binding of CD16A-SpyTag, MSLN-SpyCatcher, and MSLN×CD16A to MSLN-positive tumor cells (MKN45). CD16A-SpyTag, MSLN-SpyCatcher, and MSLN×CD16A were detected using His Tag antibody, red; cell nuclei, blue. **i** Representative confocal images showing the binding of MSLN×CD16A to MSLN-positive (NCI-N87, HGC27, and CFPAC-1) and MSLN-negative (HuH-7) tumor cells. For **d** and **f**, one-way ANOVA and Tukey’s multiple comparison test were used. Data represent mean ± SEM; *n* = 3 independent experiments. Scale bar: 50 µm. ns not significant; ∗*P* < 0.5; ∗∗*P* < 0.01; ∗∗∗*P* < 0.001; ∗∗∗∗*P* < 0.0001

To construct the bispecific cell engager MSLN×CD16A, we utilized the SpyTag/SpyCatcher protein conjugation system (Fig. 2a) to combine MSLN-SpyCatcher (for tumor localization and aggregation) and CD16A-SpyTag (for immune activation) at a molecular ratio of 1:1. Both MSLN-SpyCatcher and CD16A-SpyTag proteins were successfully induced and expressed in *Escherichia coli* Clear coli BL21 (DE3) and presented clear main bands by SDS‒PAGE (Supplementary Fig. S1a), with purities exceeding 90%, as determined by high-performance liquid chromatography (HPLC) (Supplementary Fig. S1b). Owing to potential inaccuracies in protein concentration measurements, the two proteins were mixed and coupled at different ratios, with a 1:1 molecular ratio used as the baseline. SDS‒PAGE analysis was performed to identify the optimal reaction conditions for obtaining the bispecific cell engager MSLN×CD16A (Fig. 2b, c). Flow cytometry analysis confirmed the binding of MSLN×CD16A to CD16A on CIML NK cells (Fig. 2d), with 10 µg of MSLN×CD16A reaching saturation binding to 1 × 10⁵ CIML NK cells (Supplementary Fig. S1c). Furthermore, both the flow cytometry and immunofluorescence staining results confirmed that MSLN×CD16A could specifically bind to MSLN-positive tumor cells (MKN45, NCI-N87, HGC27, and CFPAC-1) but did not bind to MSLN-negative tumor cells (MiaPaCa-2 and HuH-7) (Fig. 2e-i, Supplementary Fig. S1d). In silico analyses via the Immune Epitope Database (IEDB) suggest that the SpyTag/SpyCatcher system has low immunogenicity potential (Supplementary Fig. S1e, f).

### Bispecific MSLN×CD16A specifically enhances CIML NK effector function in MSLN-positive tumor cells

**Fig. 3:**
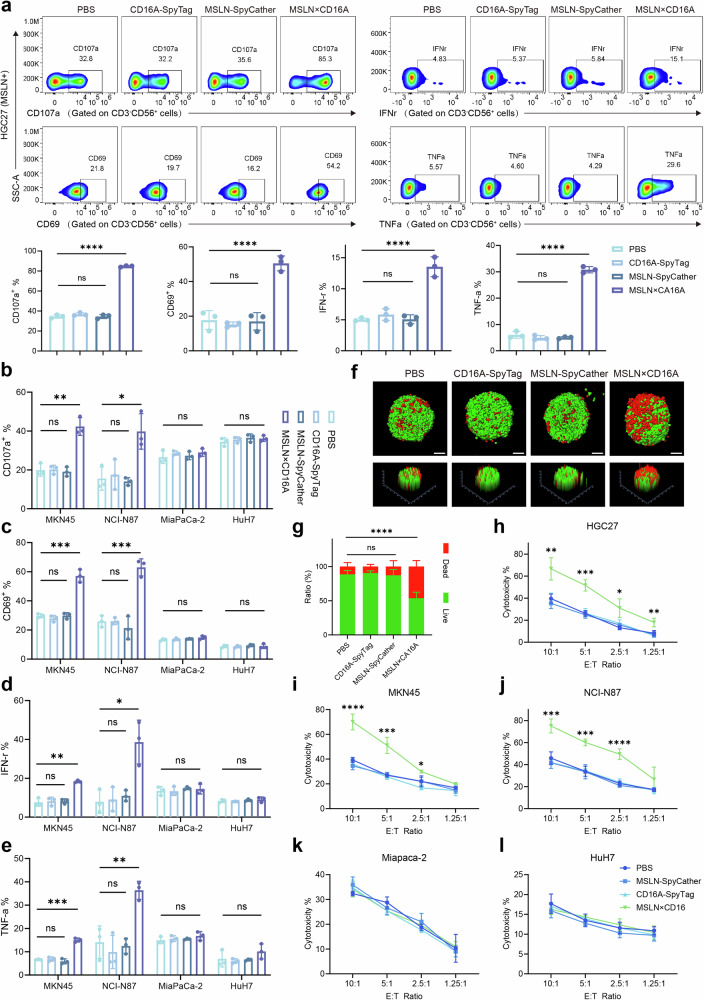
MSLN×CD16A potently induces CIML NK cell activation and depletion of MSLN-positive target cells. **a** CIML NK cells were cocultured with MSLN-positive target cells HGC27 at an E:T ratio of 5:1 for 6 h and treated with PBS, CD16A-SpyTag, MSLN-SpyCatcher, or MSLN×CD16A to assess CIML NK cell expression of CD107a (a degranulation marker), CD69, intracellular IFNγ and TNFα. Representative flow cytometry images are shown in the upper panel. **b–e** Different MSLN-positive target cells (MKN45 and NCI-N87) and MSLN-negative target cells (MiaPaCa-2 and HuH7) were cocultured and treated as described in (**a**) to evaluate CIML NK cell functions, including CD107a, CD69, intracellular TNF-α and IFN-γ, via flow cytometry. **f** CIML NK cell cytotoxicity under different treatments was evaluated via a cell viability (green)/toxicity (red) assay after coincubation with HGC27-MCSs at an E:T ratio of 10:1. Representative confocal microscopy images of HGC27-MCS cells (upper panel) and corresponding surface display images (lower panel) are shown. **g** Quantitative analysis of live/dead cells in HGC27-MCS as shown in (**f**). **h–l** CIML NK cell cytotoxicity was measured under different treatment conditions at the indicated E:T ratios. MCS multicellular spheroids. For **a–e** and **g**, one-way ANOVA and Tukey’s multiple comparison test were used. For **h–l**, two-way ANOVA and Tukey’s multiple comparison test were used. The data are presented as the means ± SEMs; *n* = 3 independent experiments. Scale bar: 100 µm. ns not significant; ∗*P* < 0.5; ∗∗*P* < 0.01; ∗∗∗*P* < 0.001; ∗∗∗∗*P* < 0.0001

To evaluate whether MSLN×CD16A induces CIML NK cell effector activation against MSLN-positive tumor cells in vitro, CIML NK cells were cocultured with various target cells for 6 h and treated with PBS or equal concentrations of the effector-end CD16A-SpyTag, target-end MSLN-SpyCatcher, or MSLN×CD16A. In the presence of MSLN-positive tumor cells (HGC27, MKN45, and NCI-N87), CIML NK cells cocultured with MSLN×CD16A presented significantly increased levels of degranulation, as assessed by CD107a expression (Fig. 3a, b). Additionally, MSLN×CD16A treatment significantly increased the production of the inflammatory cytokines IFNγ and TNFα by CIML NK cells (Fig. 3a, d, e). Furthermore, an increase in CD69, an activation marker for CIML NK cells, was observed in the MSLN×CD16A-treated group compared with the control group (Fig. 3a, c). However, this effect was not observed in CIML NK cells cocultured with MSLN-negative tumor cells (MiaPaCa-2 and HuH7) and treated with MSLN×CD16A (Fig. 3b-e).

Most importantly, MSLN×CD16A mediated potent CIML NK cell cytotoxicity. In the multicellular spheroid (MCS) model established with the human gastric cancer cell line HGC27 (Fig. 3f), tumor cells in the control group remained mostly viable, whereas the proportion of surviving tumor cells in the MSLN×CD16A-treated group was significantly reduced, with a survival rate of 53% (Fig. 3g). Moreover, when CIML NK cells were cocultured with MSLN-positive tumor cells in vitro, MSLN×CD16A induced greater cytotoxicity, especially at high E:T ratios (Fig. 3h–j). However, MSLN×CD16A-mediated killing was specific, as it did not enhance CIML NK cell cytotoxicity against MSLN-negative cells (MiaPaCa-2 and HuH7) (Fig. 3k, l). These results demonstrate that MSLN×CD16A enhances the activation of CIML NK cells and imparts target-dependent cytotoxic activity to CIML NK cells.

### Anti-MSLN CAR-like NK cells exhibit optimal activation and cytotoxic killing activity

**Fig. 4:**
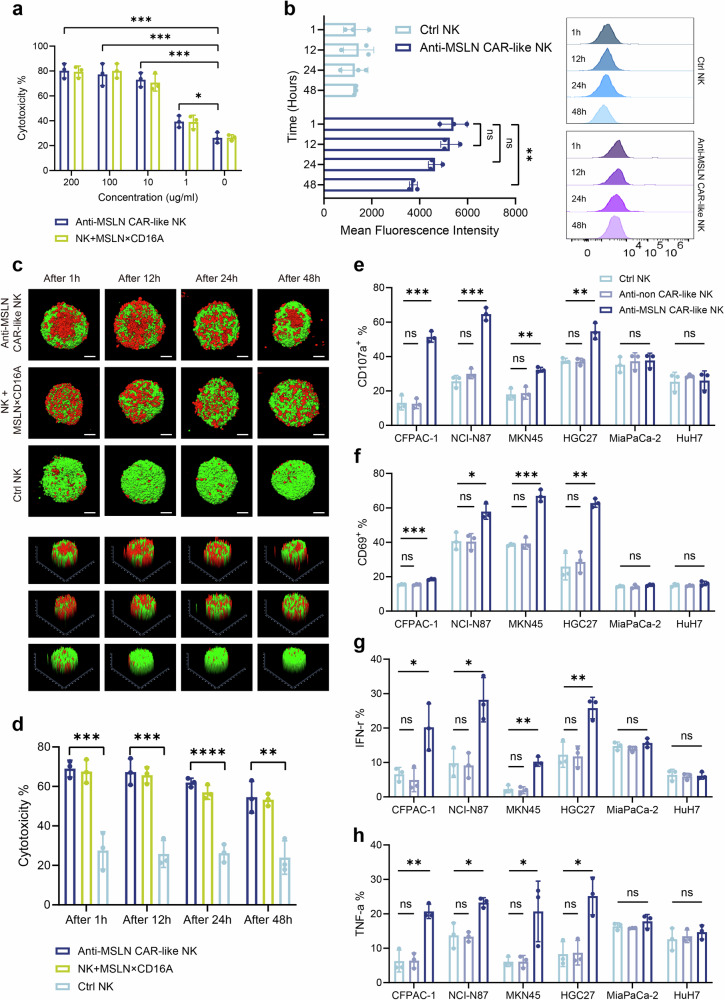
Anti-MSLN CAR-like NK cells exhibit superior activity and stable tumor cell killing capacity. **a** MSLN×CD16A was coincubated with CIML NK cells for 1 h and then washed with PBS to construct anti-MSLN CAR-like NK cells, whereas those not washed were MSLN×CD16A combined with CIML NK cell therapy (NK + MSLN×CD16A). The cytotoxicity of the two treatments against NCI-N87 (MSLN+) target cells was compared at different MSLN×CD16A concentrations, with an E:T ratio of 10:1. **b** Changes in the fluorescence intensity of 100 μg/ml MSLN×CD16A on the surface of anti-MSLN CAR-like NK (1 × 10^6^) and CIML NK (Ctrl NK) cells at different time points were evaluated by flow cytometry (left), along with representative flow cytometry images (right). **c** Comparison of the cytotoxicity for different treatments on HGC27-MCS at an E:T ratio of 10:1 over time. **d** Assessment of anti-MSLN CAR-like NK cell cytotoxicity at different time points after coincubation with NCI-N87 (MSLN+) at an E:T ratio of 5:1. **e–h** Expression of CD107a and CD69, as well as production of intracellular IFNγ and TNFα by CIML NK cells (Ctrl NK), non-targeted anti-non CAR-like NK cells, and anti-MSLN CAR-like NK cells, when cocultured with MSLN-positive target cells (CFPAC-1, NCI-N87, MKN45, and HGC27) and MSLN-negative target cells (MiaPaCa-2 and HuH7) at a 5:1 E:T ratio. The NK cells in the figures are all CIML NK cells. Ctrl NK: CIML NK cells; NK + MSLN×CD16A: MSLN×CD16A combined with CIML NK cell therapy. Two-way ANOVA and Tukey’s multiple comparison test were used for **a**, **b** and **d**. Data represent the mean ± SEM; *n* = 3 independent experiments. Scale bar: 100 µm. ns not significant; ∗*P* < 0.5; ∗∗*P* < 0.01; ∗∗∗*P* < 0.001; ∗∗∗∗*P* < 0.0001

When MSLN×CD16A is combined with CIML NK cell therapy to maximize effector activation and cytotoxic function, it is coincubated with CIML NK cells prior to infusion, allowing for the spontaneous anchoring of MSLN×CD16A on CIML NK cells, thereby constructing anti-MSLN CAR-like NK cells. To confirm the feasibility and functionality of anti-MSLN CAR-like NK cell therapy, CIML NK cells were incubated with varying concentrations of MSLN×CD16A for 1 h, followed by two washes with PBS before functional studies. Compared with MSLN×CD16A combined with CIML NK cell therapy (in vitro assay without washing after coincubation), anti-MSLN CAR-like NK cells demonstrated comparable efficiency in killing NCI-N87 (MSLN+) target cells, regardless of the MSLN×CD16A concentration (Fig. 4a). These results indicate that anti-MSLN CAR-like NK cells stably anchor MSLN×CD16A on their surface, maintaining consistent tumor cell recognition and killing capabilities.

To further evaluate the retention and activity of MSLN×CD16A on the surface of anti-MSLN CAR-like NK cells over time, 100 µg/ml MSLN×CD16A was coincubated with CIML NK cells for 1 h, washed, and cultured in medium for 1, 12, 24, or 48 h before detection. The flow cytometry results revealed that MSLN×CD16A remained on the surface of the anti-MSLN CAR-like NK cells for at least 48 h (Fig. 4b). Although the fluorescence intensity of MSLN×CD16A decreased after 48 h, likely due to partial internalization, it did not significantly affect the cytotoxic activity of the anti-MSLN CAR-like NK cells. In the HGC27-MCS model, the cytotoxic ability (approximately 50%) of the washed anti-MSLN CAR-like NK cells remained stable over time (Fig. 4c, Supplementary Fig. S1g). Similarly, the flow cytometry results revealed that, compared with CIML NK cells, anti-MSLN CAR-like NK cells exhibited significantly greater cytotoxicity against NCI-N87 (MSLN+) targets at all time points, with no significant difference from MSLN×CD16A combined with CIML NK cell therapy (Fig. 4d). These findings confirm that anti-MSLN CAR-like NK cells retain their ability to kill tumor targets over time, demonstrating the feasibility of this type of cell therapy.

Next, we evaluated the specific antitumor activity of anti-MSLN CAR-like NK cells. After 6 h of coculture, compared with CIML NK cells and anti-non CAR-like NK cells, anti-MSLN CAR-like NK cells presented significantly increased expression of CD107a and CD69, and increased IFNγ and TNFα production when cocultured with MSLN-positive target cells, including CFPAC-1, NCI-N87, MKN45, and HGC27 cells (Fig. 4e-h). In contrast, anti-MSLN CAR-like NK cells were not significantly activated by MSLN-negative cells (MiaPaCa-2 and HuH7).

### CAR-like NK cell therapy targeting MSLN demonstrates optimal tumor growth inhibition in solid tumors

**Fig. 5:**
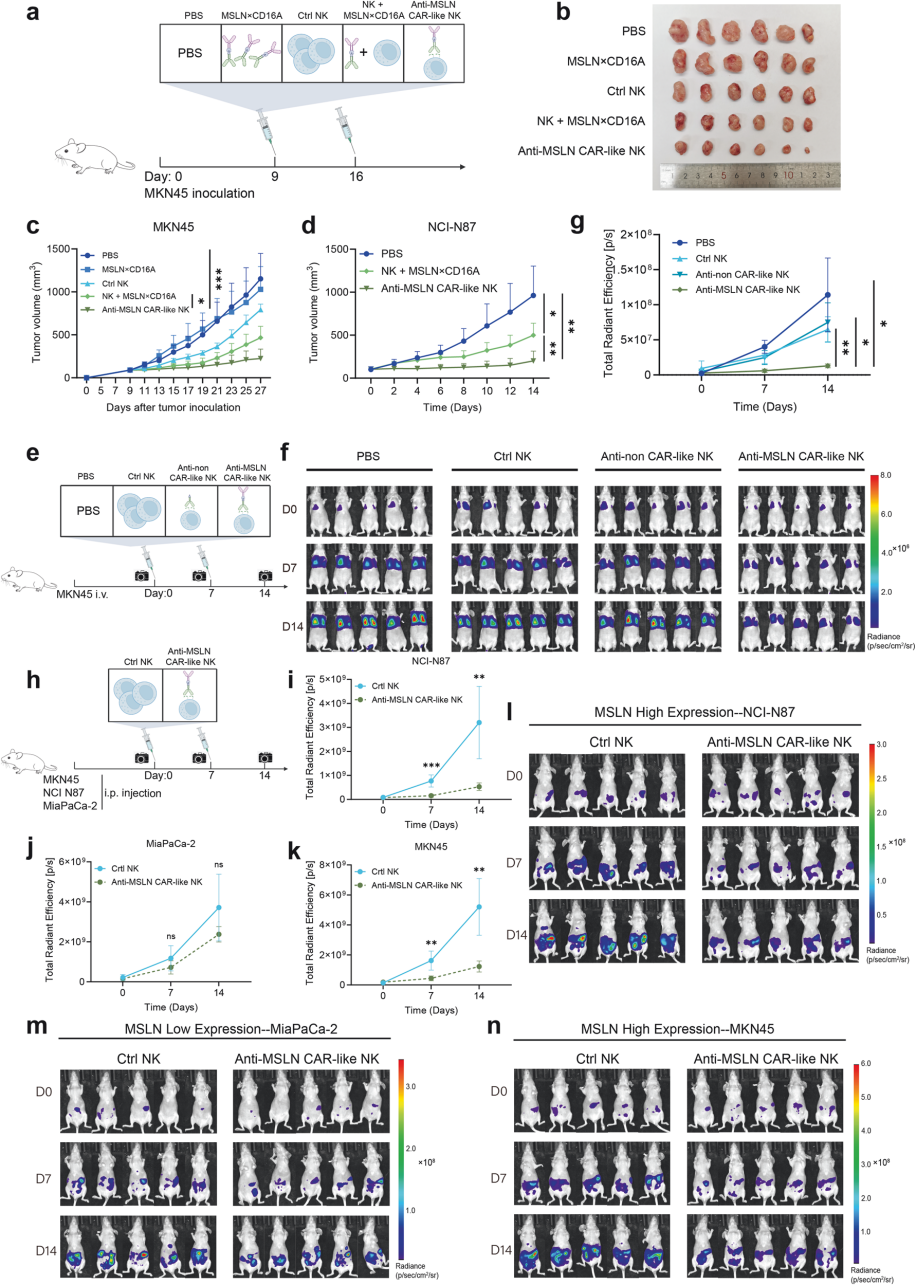
Anti-MSLN CAR-like NK cells exhibit tumor inhibition and prevent tumor cell dissemination in a preclinical xenograft mouse model. **a** Schematic diagram of the treatment regimen for MKN45 tumor-bearing BALB/c nude mice. The mice were subcutaneously implanted with 6 × 10^6^ MKN45 cells. When the tumor volume reached approximately 100 mm^3^, the mice were randomly grouped. PBS (control), MSLN×CD16A (100 µg), CIML NK (Ctrl NK, 1 × 10^7^), MSLN×CD16A combined with CIML NK cell therapy (NK + MSLN×CD16A), and anti-MSLN CAR-like NK (1 × 10^7^) were administered via tail vein injection. Recombinant human IL-2 (50,000 U) was administered intraperitoneally every other day to support the transferred NK cells. Figures were created with BioRender.com and SciDraw. **b** Tumor images harvested from different groups at the study endpoint. **c** Tumor kinetics in MKN45 tumor-bearing mice following treatment over time (*n* = 6). **d** Tumor volume curves of mice subcutaneously injected with NCI-N87 cells (*n* = 6). **e** Schematic diagram of the treatment regimen for the gastric cancer lung metastasis xenograft model. The lung metastasis model was established by injecting 3 × 10^6^ MKN45 cells into BALB/c nude mice via the tail vein. On days 0 and 7, the tumor-bearing mice were treated with PBS (control), unanchored CIML NK cells, untargeted anti-non CAR-like NK cells, or anti-MSLN CAR-like NK cells (1 × 10⁷) via intravenous reinfusion, with 50,000 U of recombinant human IL-2 every other day. The tumor burden was assessed via BLI on days 0, 7, and 14. Figures were created with BioRender.com and SciDraw. **f** Tumor burden monitored at designated time points via BLI after the corresponding treatments (*n* = 5). **g** Quantification of tumor bioluminescence images at different time points in (**f**). **h** Schematic diagram of the treatment regimen for MSLN-high- and low-expressing peritoneal disseminated tumors. The mice were intraperitoneally injected with 5 × 10⁶ MSLN-high-expressing tumor cells (MKN45, NCI-N87) or MSLN-low-expressing tumor cells (MiaPaCa-2), followed by an intraperitoneal injection of 1 × 10⁷ CIML NK cells (Ctrl NK group) or anti-MSLN CAR-like NK cells, with recombinant human IL-2 given every other day. The tumor burden was monitored at designated time points via BLI. Figures were created with BioRender.com and SciDraw. **i–k** Quantification of the bioluminescence levels of MSLN-high- and MSLN-low-expressing peritoneal disseminated tumors at different time points in (**l**–**n**). **l–n** Tumor burden was monitored via BLI in peritoneal disseminated tumor-bearing mice treated with the corresponding cells. BLI: bioluminescence imaging. Two-way ANOVA and Tukey’s multiple comparison test were used. The data represent the means ± SEMs. ns, not significant; ∗*P* < 0.5; ∗∗*P* < 0.01; ∗∗∗*P* < 0.001; ∗∗∗∗*P* < 0.0001

In vitro experiments indicate that CAR-like NK cell therapy is feasible. After washing, anti-MSLN CAR-like NK cells recognize and kill tumor cells due to the stable anchoring of MSLN×CD16A on their surface, with an efficacy comparable to that of MSLN×CD16A combined with CIML NK cell therapy. However, the antitumor efficacy of anti-MSLN CAR-like NK cells in vivo remains unclear. Therefore, we further evaluated its antitumor effect in vivo by establishing an MSLN-positive MKN45 xenograft mouse model (Fig. 5a). When the tumor volume reached 100 mm^3^, the mice were treated twice with PBS, 100 µg MSLN×CD16A, 1 × 10^7^ CIML NK cells (Ctrl NK), MSLN×CD16A combined with CIML NK cells (NK + MSLN×CD16A), or anti-MSLN CAR-like NK cells (at 7-day intervals). As shown in Fig. 5b, c, in the anti-MSLN CAR-like NK group, the tumor volume remained relatively stable at a relatively low level, whereas the tumor burden in the PBS and MSLN×CD16A groups increased dramatically. Although CIML NK cell therapy delayed tumor growth, no significant difference was observed compared to the PBS control group. Notably, anti-MSLN CAR-like NK cells demonstrated a more significant advantage in inhibiting tumor growth than MSLN×CD16A combined with CIML NK cell therapy did (*P* < 0.05), with this trend strengthening over time. These findings suggest that MSLN×CD16A combined with CIML NK cell treatment (CAR-like NK cells assembled in vivo) is not as effective as anti-MSLN CAR-like NK cells assembled in vitro in terms of antitumor effects.

To further validate the enhanced function of anti-MSLN CAR-like NK cells in a systemic environment, we conducted experiments using the NCI-N87 gastric cancer subcutaneous tumor model (Supplementary Fig. S2a). Compared with the PBS control, both types of NK cell therapy (NK + MSLN×CD16A and anti-MSLN CAR-like NK) significantly delayed tumor growth (Fig. 5d and Supplementary Fig. S2c). The average tumor weight in the anti-MSLN CAR-like NK group was 0.29 g, which was significantly lower than that in the PBS group (1.07 g) and the NK + MSLN×CD16A group (0.64 g) (Supplementary Fig. S2d). However, anti-MSLN CAR-like NK cells demonstrated a greater advantage in reducing the tumor burden (Supplementary Fig. S2e). In summary, anti-MSLN CAR-like NK cells not only demonstrate therapeutic feasibility but also exhibit unique antitumor advantages, providing new insights for tumor immunotherapy.

To assess the safety of anti-MSLN CAR-like NK cells, H&E staining of major organs, including the heart, liver, spleen, lung, and kidney, was performed on MKN45 tumor-bearing mice in each group (Supplementary Fig. S3a). No significant tissue damage or structural changes were observed. Additionally, routine blood tests and serum biochemical analysis indicated no obvious toxicity following anti-MSLN CAR-like NK cell therapy (Supplementary Fig. S3b). Furthermore, no significant body weight fluctuations were observed in the MKN45-bearing mice (Supplementary Fig. S3c) or NCI-N87-bearing mice (Supplementary Fig. S2b). These results show that anti-MSLN CAR-like NK cell therapy not only possesses excellent tumor-killing capacity but also demonstrates superior safety, offering broad potential for clinical application.

### Anti-MSLN CAR-like NK cells exhibit robust antitumor efficacy and specificity in intraperitoneal and pulmonary metastatic tumor models

To evaluate whether anti-MSLN CAR-like NK cells can inhibit distant tumor metastasis in gastric cancer, we established a lung metastasis mouse model by intravenously injecting MKN45 cells into mice (Fig. 5e). In this model, tumor cells were detected in the lungs (Fig. 5f), successfully mimicking the lung metastasis of gastric cancer. One and two weeks after tumor cell implantation, the mice were infused with two doses of PBS, 1 × 10^7^ CIML NK cells, untargeted anti-non CAR-like NK cells, or anti-MSLN CAR-like NK cells. The infused NK cells were supported by rh-IL2 (intraperitoneal injection), and tumor growth was monitored weekly via BLI (Fig. 5f). Overall, anti-MSLN CAR-like NK cells significantly suppressed tumor cells in the lungs of most mice, whereas CIML NK and anti-non CAR-like NK cells failed to effectively control tumor progression (Fig. 5f, g). In addition, treatment with anti-MSLN CAR-like NK cells was well tolerated, with body weights remaining stable across all groups (Supplementary Fig. S3d). These findings suggest that anti-MSLN CAR-like NK cells can safely and effectively inhibit the distant metastasis of MSLN-positive tumor cells.

Next, we assessed the antitumor efficacy and specificity of anti-MSLN CAR-like NK cells in a peritoneal disseminated tumor mouse model. MSLN-high-expressing tumor cells (MKN45 and NCI-N87) and MSLN-low-expressing tumor cells (MiaPaCa-2) were injected intraperitoneally into the mice (Fig. 5h). Ten days later, tumor cell proliferation in the peritoneum was detected via BLI. The mice were then divided into two experimental groups: one group received an intraperitoneal infusion of 1 × 10⁷ CIML NK cells (Ctrl NK group), and the other group was infused with 1 × 10⁷ anti-MSLN CAR-like NK cells. The results revealed that anti-MSLN CAR-like NK cells significantly inhibited the growth of MSLN-high-expressing tumor cells, whereas tumors in the control group progressed rapidly (Fig. 5i, k). BLI images revealed significant differences between the anti-MSLN CAR-like NK cell treatment group and the control group at days 7 and 14 (Fig. 5l, n). However, for mice bearing MiaPaCa-2 tumors with low MSLN expression, CAR-like NK cell therapy did not significantly slow tumor growth or disease progression (Fig. 5j, m). No toxic reactions, such as weight loss, were observed in the anti-MSLN CAR-like NK-treated mice with disseminated peritoneal tumors (Supplementary Fig. S3e). Together, our data demonstrate that anti-MSLN CAR-like NK cells exhibit specific and potent antitumor activity against MSLN-positive tumor cells in vivo.

### CAR-like NK cells engineered for tumor penetration display superior homing and infiltrative capacity

Although anti-MSLN CAR-like NK cells have demonstrated potent antitumor activity in vivo, the dense extracellular matrix and high interstitial pressure of solid tumor tissue often limit the infiltration capacity of immune cells, thereby compromising therapeutic efficacy.^9^ To enhance the penetration of anti-MSLN CAR-like NK cells into the tumor stroma and further improve antitumor efficacy, we conjugated the tumor-penetrating peptide uCendR (RPARSGR↓SAGGSVA)^17^ to the MSLN end. This modification, which is based on the common cleavage motif of urokinase-type plasminogen activator (uPA), endows anti-MSLN uCAR-like NK cells with both tumor-targeting and tissue-penetrating capabilities. Notably, uPA (PLAU) and NRP-1 transcripts are significantly upregulated across most cancer types (Supplementary Fig. S4a).

**Fig. 6:**
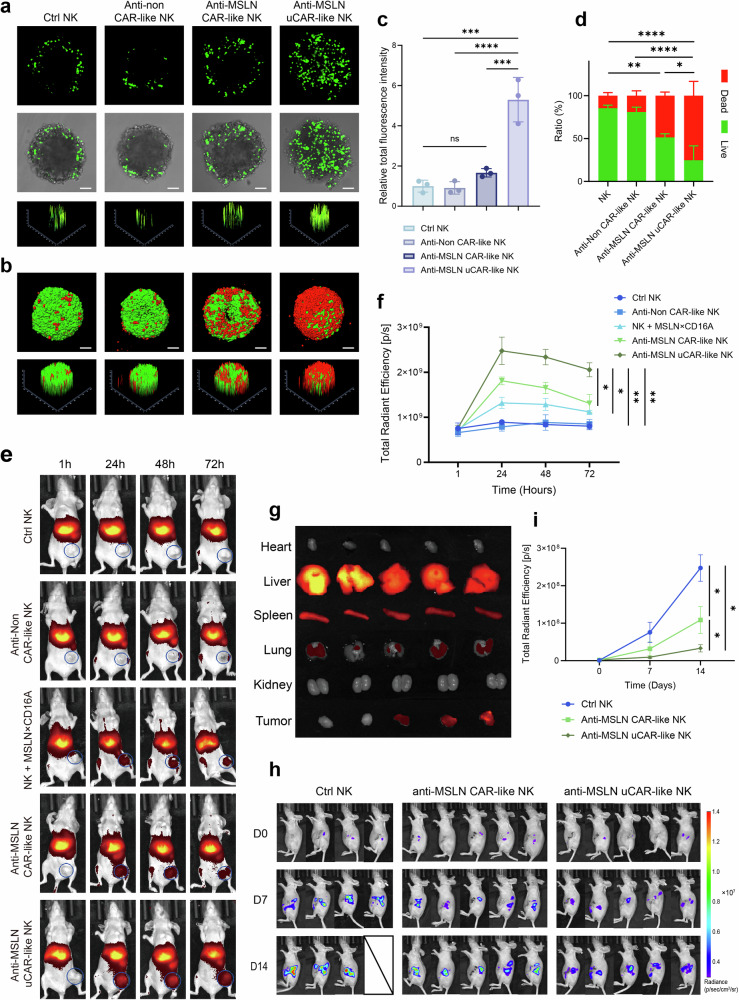
Anti-MSLN uCAR-like NK cells demonstrate superior tumor infiltration and antitumor efficacy. **a** Confocal microscopy images (upper and middle panels) and surface plot images (lower panel) showing the penetration of CFSE-labeled CIML NK, anti-non CAR-like NK, anti-MSLN CAR-like NK, or anti-MSLN uCAR-like NK into HGC27-MCSs at an E:T ratio of 10:1. **b** Evaluation of the cytotoxicity of each group in (**a**) to HGC27-MCSs. **c** Mean CFSE fluorescence intensity of HGC27-MCS in (**a**). **d** Quantification of live/dead cells in HGC27-MCS from (**b**). **e** Representative in vivo images at different time points following intravenous injection of different cell types (CIML NK, anti-non CAR-like NK, NK + MSLN×CD16A, anti-MSLN CAR-like NK, or anti-MSLN uCAR-like NK) in MKN45 subcutaneous tumor-bearing mice, with tumor locations marked by circles. **f** Time-dependent changes in the signal intensity of different cell types in the tumors shown in (**e**). **g** Ex vivo images of the heart, liver, spleen, lung, kidney, and tumor 24 h after intravenous injection. **h** In the orthotopic pancreatic cancer mouse model, CIML NK cells, anti-MSLN CAR-like NK cells, or anti-MSLN uCAR-like NK cells (1 × 10⁷) were administered intraperitoneally, with treatment repeated twice at 7-day intervals. Recombinant human IL-2 was given concurrently. The tumor burden was assessed via BLI on days 0, 7, and 14. **i** Quantification of bioluminescent tumor images at different time points, as shown in (**h**). For **c** and **d**, one-way ANOVA and Tukey’s multiple comparison test were used. For **f** and **i**, two-way ANOVA and Tukey’s multiple comparison test were used. The data represent the means ± SEMs; *n* = 3 independent experiments. Scale bar: 100 µm. ns not significant; ∗*P* < 0.5; ∗∗*P* < 0.01; ∗∗∗*P* < 0.001; ∗∗∗∗*P* < 0.0001

To investigate the tumor penetration of anti-MSLN uCAR-like NK cells in vitro, they were labeled with CFSE and coincubated with HGC27-MCSs for 6 h. As shown in Fig. 6a, CIML NK, anti-non CAR-like NK, and anti-MSLN CAR-like NK cells were confined to the periphery of the tumor spheroid, with only weak signals detected. In contrast, anti-MSLN uCAR-like NK cells actively penetrated the tumor spheroid, demonstrating significantly stronger penetration ability, which was 5.3 times greater than that of CIML NK cells, 5.8 times greater than that of anti-non CAR-like NK cells, and 3.2 times greater than that of anti-MSLN CAR-like NK cells (Fig. 6c).

In vivo experiments further validated the homing and penetration capabilities of anti-MSLN uCAR-like NK cells. In the subcutaneous tumor model of gastric cancer, anti-MSLN uCAR-like NK cells accumulated significantly at the tumor site, reaching a peak 24 h after treatment (Fig. 6e, f). In contrast, CIML NK and anti-non CAR-like NK cells presented minimal signals in the tumor region because of the lack of targeted localization. Notably, although MSLN×CD16A combined with CIML NK cells demonstrated a certain homing effect, the signals were weaker than those of anti-MSLN CAR-like NK cells assembled in vitro, potentially explaining why MSLN×CD16A combined with CIML NK cell therapy exhibited inferior antitumor efficacy in vivo. The tumors were resected and measured in vitro 24 h after infusion (Fig. 6g). Consistent with the results from whole-body imaging, the highest degree of cell accumulation at the tumor site was observed in the anti-MSLN uCAR-like NK group. Ex vivo organ imaging also revealed that the infused cells primarily accumulated in the liver and spleen, with minimal accumulation in other organs. The accumulation of anti-MSLN uCAR-like NK cells in non-tumor sites aligns with the natural homing and distribution patterns of NK cells, which are known to reside in the liver and spleen. In summary, anti-MSLN uCAR-like NK cells exhibited superior penetration and homing capabilities in MCSs and tumor tissues.

Given the superior penetration and homing properties of anti-MSLN uCAR-like NK cells, we further evaluated their antitumor efficacy. As expected, anti-MSLN uCAR-like NK cells exhibited superior antitumor efficacy (Supplementary Fig. S4b, c). Notably, anti-MSLN uCAR-like NK cells exhibited antitumor activity comparable to that of anti-MSLN CAR-like NK cells against MSLN-positive targets. This may be attributable to the absence of a penetration step during their interaction with monolayer tumor cells in vitro. We then evaluated anti-MSLN uCAR-like NK cells in the HGC27-MCS model, where their improved penetration translated into markedly enhanced tumor-targeted cytotoxicity (Fig. 6b, d). Anti-MSLN uCAR-like NK cells achieved approximately 80% cytotoxicity, which was significantly greater than that of anti-MSLN CAR-like NK cells (~50%, Fig. 6d). In the subcutaneous MKN45 (MSLN+) xenograft model, anti-MSLN uCAR-like NK cells significantly inhibited tumor growth with good tolerability (Supplementary Fig. S5). The adaptor protein used to arm anti-MSLN uCAR-like NK cells was incubated with human PBMCs for 24 h without target cells. No PBMC activation was observed, which is consistent with a favorable safety profile and a low risk of target-independent immune stimulation (Supplementary Fig. S4d). To better understand the effects of the tumor microenvironment and tumor barriers on immune cell penetration, we used a pancreatic orthotopic tumor model. In this model, MSLN-positive CFPAC-1 human pancreatic cancer cells were injected into the pancreas of mice. Three weeks after tumor cell injection, two doses of PBS, anti-MSLN CAR-like NK cells, or anti-MSLN uCAR-like NK cells (1.0 × 10⁷) were administered intraperitoneally, with rh-IL2 administered every other day. The PBS control group presented the most pronounced tumor invasion and disease progression. In contrast, anti-MSLN uCAR-like NK cells significantly inhibited tumor growth. Anti-MSLN CAR-like NK cells, which only exhibited targeted effects, tended to slow tumor growth and disease progression, but their efficacy was inferior to that of anti-MSLN uCAR-like NK cells with penetrating activity (Fig. 6h, i). Overall, our data demonstrate that anti-MSLN uCAR-like NK cells exhibit significant advantages in terms of antitumor efficacy.

## Discussion

The emergence of NK cell-based immunotherapy in recent years has introduced a new paradigm for tumor treatment. Owing to their inherent cytotoxicity, lower risk of GvHD, and reduced incidence of CRS, NK cells are ideal for the development of “off-the-shelf” allogeneic cell therapies.^7,21^ In this study, we demonstrate the potential of anti-MSLN CAR-like NK cell therapy for treating MSLN-positive solid tumors both in vitro and in vivo, supporting the translation of this approach to clinical applications.

In this study, CAR-like NK cells were developed using CIML NK cells as carriers, a differentiated state induced by short-term cytokine activation, which results in excellent cytotoxicity, degranulation, and cytokine production capabilities.^4,22^ Since 2014, the adoptive transfer of CIML NK cells has gradually emerged as an important immunotherapy for hematologic malignancies, particularly for treating relapsed/refractory acute myeloid leukemia.^6^ Currently, phase I clinical trials are evaluating the potential efficacy and safety of CIML NK cells in patients with advanced metastatic head and neck cancer (NCT04290546), advanced clear cell renal cell carcinoma and urothelial carcinoma (NCT06318871), and relapsed high-grade ovarian cancer (NCT06321484). Studies have shown that CIML NK cells, compared with conventional NK cells, express higher levels of activation receptors, exhibit increased expression of cell cycle-related genes and heme synthesis genes, and lower expression of inhibitory genes.^23^ The increased heme synthesis may be crucial for the enhanced antitumor activity of CIML NK cells, as heme plays an essential role in energy and iron metabolism. CIML NK cells can also be genetically modified with chimeric antigen receptors (CARs), which exhibit greater functional activity than conventional CAR NK cells.^24^ Studies have shown that CD19-CAR CIML NK cells exhibit stronger cytotoxic activity against NK-resistant lymphoma cell lines than do CD19-CAR NK cells in vitro.^24^ Tarannum M’s team reported that CAR CIML NK cells targeting MSLN showed promising activity in ovarian cancer.^23^

We employed the SpyTag/SpyCatcher system to construct the bispecific cell engager MSLN×CD16A and to develop a nongenetically engineered CAR-like NK cell therapy. This “molecular superglue” confers modular universality on the CAR-like NK cell platform. By substituting tumor-binding domains, the platform can be retargeted to additional solid tumor antigens, thereby broadening the population of patients who may benefit. The SpyTag/SpyCatcher system is a widely used protein‒protein interaction system with documented applications in vaccine design, nanomaterial engineering, and enzyme stabilization.^20,25^ However, the system originates from bacterial components and may carry an immunogenicity risk. In preclinical studies of engineered immune cell therapies, there has been no specific evidence of adverse immunogenicity attributed to SpyTag/SpyCatcher.^26–28^ In our study, safety evaluations revealed no evidence suggestive of immunogenicity. IEDB-based in silico analyses indicated low immunogenic potential for SpyTag/SpyCatcher, but the prediction is constrained by dependence on sequence homology instead of functional assays. Accordingly, future work will include repeat-dose studies in non-human primate models with anti-drug antibody (ADA) measurements and cell-based neutralizing antibody assays.

CIML NK cells have robust CD16 expression and exhibit enhanced antibody-dependent cellular cytotoxicity,^29^ indicating that they can be targeted via therapeutic monoclonal antibodies or bispecific/trispecific NK cell engagers. In this study, we precomplexed CIML NK cells with MSLN×CD16A to confer the ability to target MSLN-positive tumor cells, thereby avoiding genetic engineering and vector-mediated gene transfer. This approach simplifies the production process and reduces costs. The constructed anti-MSLN CAR-like NK cells exhibited excellent tumor control both in vitro and in vivo. Studies have shown that precomplexing umbilical cord blood-derived NK cells with AFM13 (a tetravalent bispecific antibody targeting CD30/CD16A) can generate stable CAR-like NK cells with potent cytotoxicity against CD30-positive lymphoma.^11^ Compared with the combined infusion of AFM13 and NK cells as two separate products, this CAR-like NK cell therapy strategy can avoid the dilution and uptake of AFM13 by endogenous NK cells, thereby avoiding the impact of these endogenous NK cells with low cytotoxic activity on efficacy.^11^ A phase I/II clinical study of relapsed/refractory CD30-positive lymphoma (NCT04074746) has shown the significant efficacy of this therapy. Among 42 patients with relapsed/refractory lymphoma, the overall response rate was 92.9%, the complete response rate was 66.7%, and the 2-year overall survival rate was 76.2%, with no serious adverse effects, such as CRS, neurotoxicity, or GvHD observed.^30^

In this study, we demonstrated that anti-MSLN CAR-like NK cells exhibit potent and specific activity against MSLN-positive tumor cell lines and effectively inhibit tumor growth in xenograft mouse models. The tumor cell surface antigen MSLN, which is highly expressed in mesothelioma, pancreatic cancer, gastric cancer, ovarian cancer and other cancers,^13^ plays a crucial role in promoting tumor cell proliferation, local invasion, metastasis, and resistance to cytotoxic-induced apoptosis.^31–33^ The overexpression of MSLN activates key signaling pathways, including the Akt/PI3K/NF-κB pathway, and upregulates Mcl-1, contributing to cancer cell proliferation, invasion, and metastasis.^14,34^ Additionally, the high-affinity interaction between MSLN and CA125 promotes tumor cell adhesion and metastasis.^35^ Multiple studies have indicated that MSLN is an attractive therapeutic target for CAR-T and CAR-NK cell-based immunotherapy, as well as for antibody‒drug conjugates in various solid tumors. The FDA orphan drug designation for BZD1901, a CAR-T cell therapy targeting MSLN, further demonstrates the promise of MSLN-targeted therapies in cancer treatment. Compared with these modalities, our strategy incorporates three key innovations. First, genetic engineering and vector-mediated transduction are not needed. Instead, CAR-like activity is directly induced, which streamlines manufacturing and may reduce costs. Second, a modular platform is established via the SpyTag/SpyCatcher conjugation system, allowing the exchange of tumor-binding domains to increase eligibility. Third, the uCendR module is incorporated to confer intratumor penetration.

CAR-NK cell therapy has broad potential in cancer immunotherapy, especially in the treatment of solid tumors.^2,36^ However, one major challenge faced by CAR-NK cell therapy in solid tumors is the limited ability of NK cells to infiltrate the tumor, which limits the clinical outcomes. Increasing NK cell infiltration into solid tumors is crucial for the success of cancer immunotherapy.^37^ In our study, anti-MSLN CAR-like NK cells induced tumor suppression and inhibited tumor cell dissemination in xenograft mouse models. However, in a pancreatic orthotopic tumor model, which better mimics the tumor microenvironment, the same dose of anti-MSLN CAR-like NK cells had limited therapeutic efficacy. In contrast, anti-MSLN uCAR-like NK cells, which possess tumor-penetrating capabilities, enhanced NK cell infiltration into the tumor tissue, resulting in tumor regression. These findings suggest that the integration of the tumor-penetrating peptide uCendR can further enhance the antitumor activity of anti-MSLN CAR-like NK cells. The adaptor-fused uCendR peptide remains inert in circulation and becomes active only after cleavage by uPA, thereby promoting deeper intratumor penetration of anti-MSLN uCAR-like NK cells. uPA is present at low basal levels in some physiological settings (such as wound healing and tissue remodeling), increasing the theoretical risk of off-target activation. We consider this risk manageable given the greater expression of uPA/uPAR and NRP-1 in tumors than in normal tissues, the requirement for local NRP-1 colocalization for activation, and our safety observations. Tumor-penetrating peptides possess several favorable translational characteristics, such as a lack of species specificity, low immunogenicity, and cost-effective production.^38^ They have been used to increase the penetration of drugs, nanocarriers, and immune cells.^39,40^ Our previous studies revealed that T cells and CIML NK cells modified with the tumor-penetrating peptide iRGD exhibit significantly improved intratumor infiltration and excellent antitumor activity.^41,42^ Tumor-penetrating peptides are currently being evaluated in multiple clinical trials (NCT06592664, NCT06261359, NCT05121038, NCT05712356, NCT05042128, etc.) as co-administered enhancers for standard-of-care cancer therapies. In a first-in-human, open-label, multicenter, phase 1 study (NCT03517176), the tumor-penetrating peptide CEND combined with nab-paclitaxel and gemcitabine showed acceptable safety and tolerability in patients with metastatic pancreatic ductal adenocarcinoma.^43^ To date, no peptide-specific immunogenicity signal has been reported. No evidence of immunogenicity was detected in this study. Subsequent studies will assess immunogenicity through ADA measurements and neutralizing antibody assays. Other strategies to improve CAR-NK cell therapy for solid tumors include co-expressing immune-stimulatory cytokines,^44–46^ modifying NK cells to overcome TME-mediated immune suppression,^47^ and optimizing CAR constructs to enhance NK cell signaling.^37^ These strategies will be tested in future studies to further enhance the efficacy of anti-MSLN uCAR-like NK cells for solid tumors.

Despite these promising results, our study has several limitations. First, we used a BALB/c nude model, which lacks key immune compartments that can contribute to an immunosuppressive TME. Consequently, this model fails to fully recapitulate the complexity of human solid tumors, including stromal–immune crosstalk and metastatic behavior. Second, we used immunodeficient nude mice to evaluate the short-term effects of anti-MSLN CAR-like NK cell therapy. However, long-term antitumor responses, the development of resistance, and off-target effects remain unclear. Third, we focused on MSLN-positive tumor cell lines (such as HGC27, MKN45, NCI-N87, and CFPAC-1) to assess antitumor efficacy, concentrating on gastrointestinal tumors, which may limit the generalizability of our findings. Compared with the cell line-derived xenografts used in this study, PDX models better recapitulate the human tumor architecture, heterogeneity, and features of the tumor microenvironment, such as the extracellular matrix and stromal components. Future studies will evaluate the long-term efficacy and potential risks of anti-MSLN uCAR-like NK cells in MSLN-positive PDX models to optimize therapy and facilitate clinical translation.

In conclusion, we demonstrated the feasibility and efficacy of anti-MSLN CAR-like NK therapy, which is based on CIML NK cells, against MSLN-positive tumors both in vitro and in vivo. Anti-MSLN uCAR-like NK cells, endowed with tumor-penetrating capabilities, are able to infiltrate tumor tissues and induce tumor regression, providing a solid foundation for the clinical application of immunotherapies targeting MSLN-positive solid tumors.

## Materials and methods

### Ethics approval and consent to participate

The blood collection procedure was carried out in accordance with the guidelines verified and approved by the ethics committee of Drum Tower Hospital. All the donors signed an informed consent for scientific research statements. All animal procedures were carried out in compliance with the guidelines set by the Animal Care Committee at Drum Tower Hospital (Nanjing, China). The ethics committee of Drum Tower Hospital approved all the experiments in this study (Approval ID: 2023AE01097).

### Cell lines

The human gastric cancer cell lines MKN45, HGC27, and NCI-N87; the human liver cancer cell line HuH7; the human myeloid leukemia cell line K562; and the human pancreatic cancer cell lines CFPAC-1 and MiaPaCa-2 were purchased from the Cell Bank of the Shanghai Institute of Biochemistry and Cell Biology. The gastric cancer cell lines and K562 cells were cultured in Roswell Park Memorial Institute (RPMI) 1640 medium supplemented with 10% fetal bovine serum (FBS), 100 U/mL penicillin, and 100 µg/mL streptomycin under standard conditions of 37°C and 5% CO₂. The other cell lines were cultured in DMEM supplemented with 10% FBS. K562 feeder cells (K562-CD48-41BBL-mbIL-21) were prepared as previously described for the in vitro expansion of NK cells.^48^ The identities of all the cell lines were confirmed by phenotyping or genotyping, and regular mycoplasma testing was performed to ensure the absence of mycoplasma contamination.

### Acquisition of the bispecific cell engager MSLN×CD16A

The design and construction of MSLN-SpyCatcher were as follows: His tag-MSLN DARPin-SpyCatcher-MSLN DARPin, and CD16A-SpyTag was designed as His tag-CD16A DARPin-SpyTag-CD16A DARPin. The corresponding DNA fragments were synthesized by GenScript (Nanjing) and cloned into the pET-21b bacterial expression vector. Enzyme digestion and DNA sequencing confirmed the correct insertion. The recombinant protein was expressed in *Escherichia coli* Clear coli BL21 (DE3), and the bacterial pellets were collected after induction and lysed via sonication. The proteins were purified via an AKTA system (GE Healthcare, CT, USA) with a HisTrap HP column. The target protein was confirmed by 15% sodium dodecyl sulfate‒polyacrylamide gel electrophoresis (SDS‒PAGE), and the purity was tested via HPLC. The purified protein was dialyzed against phosphate-buffered saline (PBS) and sterilized by filtration through a 0.22 µm membrane. The protein concentration was determined via a BCA protein assay kit (Thermo Fisher Scientific, Massachusetts, USA). Owing to the limited accuracy of protein concentration measurements, MSLN-SpyCatcher and CD16A-SpyTag were mixed at different ratios based on a 1:1 molar ratio to determine the optimal conjugation conditions for generating MSLN×CD16A. To enhance tumor penetration, the tumor-penetrating peptide uCendR (His tag-MSLN DARPin-SpyCatcher-MSLN DARPin-uCendR) was integrated into the design of MSLN-SpyCatcher, and the same approach was used to construct MSLN×CD16A containing the tumor-penetrating sequence.

### CAR-like NK cell generation

Ficoll density gradient centrifugation was used to isolate mononuclear cells from cord blood (CB). The cells were preactivated with rhIL-12 (10 ng/mL), rhIL-15 (50 ng/mL), and rhIL-18 (50 ng/mL), and after 16 h, the cytokines were removed via washes with PBS, which induced the differentiation of CB NK cells into memory-like (ML) NK cells. Inactivated K562 cells were used as feeder cells for the expansion of CIML NK cells in vitro. CIML NK cells were cocultured with MSLN×CD16A for 1 h and then washed twice with PBS to generate anti-MSLN CAR-like NK cells. The washed CAR-like NK cells were cultured in the presence of IL-2 at 37 °C for 12 to 48 h, followed by incubation with a His tag antibody to validate the stability of MSLN×CD16A binding to CIML NK cells via flow cytometry.

### Flow cytometry analysis

Flow cytometric analysis was performed via the following fluorochrome-conjugated antibodies: anti-CD3-FITC (UCHT1, BD Biosciences), anti-CD56-BB700 (NCAM16.2, BD Biosciences), anti-CD69-BV421 (FN50, BD Biosciences), anti-CD107a-PE (H4A3, BD Biosciences), anti-IFNγ-PE (B27, BD Biosciences), and anti-TNFα-APC (MAb11, BD Biosciences). All the samples were suspended in flow cytometry (FACS) staining buffer and incubated with the indicated antibodies for 30 min in the dark at 4 °C. The samples were then washed twice and resuspended in FACS buffer before analysis. Flow cytometry data were collected via a CytoFLEX flow cytometer (Beckman Coulter, USA) and analyzed with FlowJo V.10.9 software.

### Functional evaluation of degranulation (CD107a), activation (CD69), and cytokine production (IFNγ and TNFα)

For CD107a and CD69 assessment, CIML NK cells were cocultured with target cells (E:T ratio of 5:1) and treated with PBS, CD16A-SpyTag, MSLN-SpyCatcher, or MSLN×CD16A (all at 100 µg/mL), followed by staining with anti-CD107a-PE. After 1 h, GolgiStop (1:1500, BD Biosciences) and GolgiPlug (1:1000, BD Biosciences) were added, and the cells were incubated for 5 h. After being washed with PBS, the cells were stained with live/dead dye, anti-CD3-FITC, anti-CD56-BB700, and anti-CD69-BV421.

To evaluate cytokine production (IFN-γ and TNF-α), the cells were cocultured as described for CD107a measurement. After 1 h, GolgiStop and GolgiPlug were added, and the cells were incubated for 5 h. After being washed and stained with live/dead dye and surface antibodies, the cells were fixed, permeabilized, and stained with anti-IFNγ-PE and anti-TNFα-APC. Finally, the stained cells were analyzed via a CytoFLEX flow cytometer. The data are presented as the percentage of NK cells positive for CD107a, CD69, and cytokine production, which were calculated by gating on CD3^-^CD56^+^ NK cells.

### Cytotoxicity assay

The cytotoxicity of CIML NK cells was assessed via the carboxyfluorescein succinimidyl ester and propidium iodide (CFSE/PI) staining method. Tumor target cells were first labeled with 2 µM CFSE (Invitrogen, USA) for 10 min at 37°C and then washed. CIML NK cells were cocultured with CFSE-labeled target cells at different effector-to-target cell ratios (E:T ratios) for 6 h. Afterward, the target cells were stained with PI (Biyuntian, China), and cytotoxicity was evaluated.

### Functional evaluation of CIML NK cells in multicellular spheroids (MCSs)

Human gastric cancer cells HGC27 were seeded into 96-well transparent round-bottom, ultralow attachment microplates (Corning, USA), each well containing RPMI 1640 medium supplemented with 10% FBS (1000 cells/well). The plate was incubated at 37 °C with 5% CO_2_ until the MCS diameter reached ~400 μm. Only uniform and dense tumor spheroids were selected for further analysis.

For the MCS cytotoxicity assays, CIML NK cells were added to the MCSs at an E:T ratio of 10:1, along with MSLN×CD16A or control reagents. After 6 h of incubation at 37 °C, the MCSs were treated with Calcein AM and EthD-III (viability/cytotoxicity assay kit, Biotium, USA). After washing with PBS, the MCSs were placed into confocal culture dishes (In Vitro Scientific, Austria) and imaged under a Leica TCS SP8 confocal microscope (Leica, Germany). The EthD-III (red) signals indicate dead cells, whereas the Calcein-AM (green) signals indicate live cells.

For penetration assessment, CFSE-labeled CIML NK cells, anti-MSLN CAR-like NK cells, or anti-MSLN uCAR-like NK cells were added to MCSs at an E:T ratio of 10:1. After 6 h of incubation at 37 °C, the MCSs were washed to remove free NK cells, fixed with 4% paraformaldehyde, and imaged via a Leica TCS SP8 confocal microscope. Images were acquired at the mid-plane of the spheroids and analyzed via ImageJ software.

### Xenograft mouse models

All animal experiments were approved by the Ethics Committee of Nanjing Drum Tower Hospital and were conducted in compliance with the guidelines set by the Animal Protection Committee of Nanjing Drum Tower Hospital. The researchers did not implement blinding in the animal experiments. Efforts were made to reduce the number of animals used and minimize animal suffering. The mice were randomly grouped on the basis of age and body weight. For the subcutaneous tumor model, 6–8-week-old female BALB/c nude mice or NSG mice were subcutaneously injected with 6 × 10⁶ MKN45 or NCI-N87 cells. The lung metastasis xenograft model was established by injecting 3 × 10⁶ MKN45 cells through the tail vein of 6–8-week-old female BALB/c nude mice. For the peritoneal metastatic tumor model, 5 × 10⁶ MKN45, NCI-N87, or MiaPaCa-2 cells were intraperitoneally injected into 6–8-week-old female BALB/c nude mice. To establish the orthotopic pancreatic cancer model, 7.5 × 10⁶ CFPAC-1 cells were orthotopically injected into the pancreas of 6–8-week-old female BALB/c nude mice. Bioluminescence imaging was performed to confirm tumor implantation before any treatment, ensuring similar tumor burdens across all the mice.

### In vivo real-time near-infrared fluorescence imaging

To evaluate the tumor-targeting efficiency of anti-MSLN uCAR-like NK cells in tumor-bearing mice, 1 × 10⁷ DiR (Bridgen, China)-labeled anti-MSLN uCAR-like NK cells were intravenously injected into MKN45 subcutaneous tumor-bearing mice. At the designated time points, the mice were anesthetized and scanned via the IVIS Lumina III system (PerkinElmer, Massachusetts, USA). For organ and tissue imaging, the mice were euthanized under deep anesthesia, and the tumors and major organs (heart, liver, spleen, lung, and kidney) were collected and imaged.

### In vivo antitumor efficacy and safety

For the subcutaneous tumor model, when the tumor volume reached ~100 mm³, the mice were randomly grouped and treated with PBS, MSLN×CD16A (100 µg), CIML NK (1 × 10⁷), MSLN×CD16A + CIML NK, or anti-MSLN CAR-like NK (1 × 10⁷) via tail vein injection, with two injections administered at 7-day intervals. Additionally, recombinant human IL-2 (50,000 U) was injected intraperitoneally every other day to support the adoptive transfer of NK cells. Tumor sizes were measured with calipers every two days, and tumor volumes were estimated via the following formula: length × width² × 0.5. For safety evaluation, peripheral blood was collected from tumor-bearing mice to assess blood parameters as well as liver and kidney function. Major organs were harvested after euthanasia, fixed in 4% paraformaldehyde, sectioned, and stained with hematoxylin and eosin (H&E).

For the MKN45 lung metastatic xenograft model, tumor-bearing mice were randomly assigned to four groups and treated with PBS, CIML NK, anti-non CAR-like NK, or anti-MSLN CAR-like NK cells (1 × 10⁷) on days 0 and 7, concurrently with recombinant human IL-2 administered every other day. The tumor burden was monitored at days 0, 7, and 14 via the IVIS Lumina III system (PerkinElmer, Massachusetts, USA).

For peritoneal metastatic tumor treatment, CIML NK or anti-MSLN CAR-like NK cells (1 × 10⁷) were intraperitoneally injected every 7 days for two doses, with recombinant human IL-2 given every other day. Mouse body weight was measured every two days.

For the orthotopic pancreatic tumor model, 1 × 10⁷ CIML NK, anti-MSLN CAR-like NK, or anti-MSLN uCAR-like NK cells were injected intraperitoneally every seven days for two doses, along with recombinant human IL-2.

### Statistical analysis

Statistical analysis and graphing were performed via GraphPad Prism 10 (San Diego, California, USA). All the data are presented as the means ± SDs. For comparisons between two groups, Student’s *t* test was used; for comparisons involving more than two groups, one-way analysis of variance (ANOVA) was applied. Tumor burden comparisons were performed via two-way ANOVA with Tukey’s multiple comparison test. *P* values < 0.05 were considered statistically significant.

## Supplementary information

Supplementary Materials

## Data Availability

All data supporting the findings of this study are available within the article and its supplementary information.
