# Supplementary material for: Anti-MSLN chimeric antigen receptor-like NK cell therapy with tumor-penetrating capacity (uCAR-like NK) for solid tumors
Source: Signal Transduct Target Ther. 2025 Dec 23;10:425. doi: 10.1038/s41392-025-02524-0 (PMC12727736; doi:10.1038/s41392-025-02524-0)
Supplement: Supplementary file 1 — Supplementary Materials [file 41392_2025_2524_MOESM1_ESM.docx]

Supplementary Materials for

Anti-MSLN Chimeric Antigen Receptor-like NK Cell Therapy with Tumor-Penetrating Capacity (uCAR-like NK) for Solid Tumors

Mengchao An^1, 2^; Ying Wang^2^; Jie Shao^2^; Siwen Wu^2^; Jiayao Yan^2^; Yuxiang Li^2^; Liqing Zhong^2^; Jingyi Guo^2^; Tianran Chen^2^; Manman Tian^2^; Qin Liu^1, 2*^; Rutian Li^2*^; Baorui Liu^1, 2*^

Correspondence to: Qin Liu ([liuqin@nju.edu.cn](mailto:liuqin@nju.edu.cn))；Rutian Li ([rutianli@nju.edu.cn](mailto:rutianli@nju.edu.cn))；Baorui Liu (baoruiliu@nju.edu.cn)

**This PDF file includes:**

Figures. S1 to S6

**Figure. S1.**

**Expression and purification of MSLN-SpyCatcher and CD16A-SpyTag**

**a.** SDS-PAGE analysis of MSLN-SpyCatcher and CD16A-SpyTag. Lane 1, protein ladder; Lane 2, supernatant of ultrasonic lysate; Lane 3, precipitate of ultrasonic lysate; Lane 4, non-target protein during purification; Lane 5, purified target protein.

**b.** Purity of MSLN-SpyCatcher (upper panel) and CD16A-SpyTag (lower panel) was assessed by HPLC.

**c.** Flow cytometric analysis of the mean fluorescence intensity of CIML NK cells co-cultured with MSLN×CD16A at different concentrations (0, 0.08, 0.4, 2, 10, 50, 250 μg/mL).

**d.** Representative confocal images showing binding of PBS control to MSLN-positive (NCI-N87, HGC27, and CFPAC-1) and MSLN-negative (HuH7) tumor cells.

**e.** B-cell epitope prediction of MSLN-SpyCatcher via Bepipred.

**f.** B-cell epitope prediction of CD16A-SpyTag via Bepipred.

**g.** Quantitative analysis of live/dead cells in HGC27-MCS in Fig. 3c.

Data represent the mean ± SEM; n = 3 independent experiments. Scale bar: 50 µm. ns, not significant; ∗P < 0.5; ∗∗P < 0.01; ∗∗∗P < 0.001; ∗∗∗∗P < 0.0001.

**Figure. S2.**

**Anti-MSLN CAR-like NK cells exhibit anti-tumor activity against NCI-N87 gastric cancer cell xenografts in vivo**

**a.** Schematic diagram of the treatment regimen for NCI-N87 tumor-bearing BALB/c nude mice. Mice were subcutaneously implanted with 6×10^6^ NCI-N87 cells and randomly divided into groups when the tumor volume reached approximately 100 mm^3^. Mice were treated with PBS (control), MSLN×CD16A combined with CIML NK cells (NK + MSLN×CD16A), or anti-MSLN CAR-like NK cells (1 × 10⁷) via tail vein injection, concurrently administered with recombinant human IL-2 (50,000 U) every other day. The figure was created with BioRender.com and SciDraw.

**b.** Body weight measurements at designated time points in each group (n = 6).

**c.** Images of tumors harvested from different groups at the study endpoint.

**d.** Weight of harvested tumors as shown in (c).

**e.** Separated spider plots depicting tumor kinetics over time for each treatment group.

Data represent the mean ± SEM. ns, not significant; ∗P < 0.5; ∗∗P < 0.01; ∗∗∗P < 0.001; ∗∗∗∗P < 0.0001.

**Figure. S3.**

**Safety of Anti-MSLN CAR-like NK Cell Therapy**

**a.** Hematoxylin and eosin (H&E) staining of major organs in MKN45 subcutaneous tumor-bearing mice following different treatments.

**b.** Blood routine and serum biochemical parameters for liver and kidney function in MKN45 subcutaneous tumor-bearing mice after treatment.

**c.** Body weight changes in MKN45 subcutaneous tumor-bearing mice at designated time points (n = 6).

**d.** Body weight changes in gastric cancer lung metastasis mice at designated time points (n = 5).

**e.** Body weight changes in peritoneal disseminated tumor-bearing mice at designated time points (n = 5).

Data represent the mean ± SEM. Scale bar: 250 µm. ns, not significant; ∗P < 0.5; ∗∗P < 0.01; ∗∗∗P < 0.001; ∗∗∗∗P < 0.0001.

**Figure. S4.**

**a.** Differential expression of PLAU (left) and NRP1 (right) between tumor and matched adjacent normal tissues across diverse TCGA cancers was analyzed with TIMER2.0. Tumors and adjacent tissues are colored in red and blue, respectively.

**b.** Expression of CD107a and CD69, as well as production of intracellular IFNγ and TNFα by CIML NK cells (Ctrl NK), anti-non CAR-like NK cells, anti-MSLN CAR-like NK cells, and anti-MSLN uCAR-like NK cells, when co-cultured with MSLN-positive target cells (NCI-N87) at a 5:1 E:T ratio.

**c.** The effector cells were co-cultured with target cells (NCI-N87) at the indicated E:T ratios.

**d.** Different adaptor proteins were incubated with human PBMCs for 24 h in the absence of target cells, and cytokines were quantified in the supernatant.

Data represent the mean ± SEM; n = 3 independent experiments. ns, not significant; ∗P < 0.5; ∗∗P < 0.01; ∗∗∗P < 0.001; ∗∗∗∗P < 0.0001.

**Figure. S5.**

**Anti-MSLN uCAR-like NK cells exhibit anti-tumor activity against MKN45 gastric cancer cell xenografts in vivo**

**a.** In vivo images of MKN45 tumor-bearing mice on day 21. NSG mice were subcutaneously inoculated with 6×10^6^ MKN45 cells and randomly divided into groups when the tumor volume reached approximately 100 mm^3^. Mice were treated with PBS, anti-MSLN CAR-like NK cells, or anti-MSLN uCAR-like NK cells (1 × 10⁷) via tail vein injection, with recombinant human IL-2 (50,000 U) administered every other day. White circles indicate tumors.

**b.** Ex vivo tumor images from different treatment groups at day 21 after inoculation.

**c.** Tumor volume curves of NSG mice subcutaneously injected with MKN45 (n = 6).

**d.** Body weight measurements at designated time points in each group (n = 6).

**e.** Separated spider plots depicting tumor kinetics over time for each treatment group in NSG mice bearing MKN45 tumors.

**f.** H&E staining of major organs in MKN45 tumor-bearing mice following different treatments.

**g.** Serum biochemistry of liver and kidney function after treatment in subcutaneous tumor-bearing NSG mice.

Data represent the mean ± SEM. Scale bar: 100 µm. ns, not significant; ∗P < 0.5; ∗∗P < 0.01; ∗∗∗P < 0.001; ∗∗∗∗P < 0.0001.

**Figure. S6.**

**a-b.** Gating strategy for Fig. 2 and Fig. 3
